# Calibration models database of near infrared spectroscopy to predict agricultural soil fertility properties

**DOI:** 10.1016/j.dib.2020.105469

**Published:** 2020-04-08

**Authors:** Agus Arip Munawar, Yuswar Yunus, Devianti, Purwana Satriyo

**Affiliations:** a Department of Agricultural Engineering, Syiah Kuala University, Banda Aceh, Indonesia; b Agricultural Mechanization Research Centre, Syiah Kuala University, Banda Aceh, Indonesia

**Keywords:** Soil, Calibration model, Datasets, NIRS, Prediction

## Abstract

Presented paper describes spectroscopic dataset and calibration models database of near infrared spectroscopy (NIRS) used to predict agricultural soil fertility properties. Near infrared spectra data in form of absorbance spectrum were acquired in wavelength range from 1000 to 2500 nm for a total of 40 bulk soil samples amounted of 10 g per each bulk. Soil fertility properties, presented as soil nitrogen (N), phosphorus (P). potassium (K), soil pH, magnesium (Mg) and calcium (Ca), were measured by means of wet chemical analysis. Calibration models, used to predict those soil fertility parameters were developed using two different regression algorithms namely principal component regression (PCR) and partial least square regression (PLSR) respectively. Prediction performance can be evaluated and justified by looking their statistical indicators: correlation of determination (R^2^), correlation coefficient (r), root mean square error (RMSE) and residual predictive deviation (RPD). Spectra data can also be corrected in order to improve and enhance prediction performance. Obtained NIRS dataset and models database can be used as a rapid and simultaneous method to determine agricultural soil fertility properties.

Specifications table

**Table:** 

Subject	Agricultural and Biological Sciences Soil sciences
Specific subject area	Spectroscopy, non-invasive and rapid method for soil fertility properties determination
Type of data	Table Graph Spectroscopic data
How data were acquired	Spectral datasets of soil samples were acquired using a benchtop Fourier transform near infrared (NIR) spectroscopy (*Thermo Nicolet Antaris II*). The instrument was controlled using integrated software: Thermo Integration® and Thermo Operation®. The light source of halogen lamp irradiated soil samples from down to up through a quartz window (1 cm of diameter), which was embedded in the top of the NIR instrument. Soil sample was packed in a sample cup which was fixed on the quartz window by swivel bracket. During spectra data acquisition, sample cup was spinning around slowly in order to obtain the averaged spectrum of each soil sample. Background spectra correction was taken once every 10 sample acquisitions. The spectral resolution was 8 cm^−1^ and optical gain was set to 4x. Spectral data was collected and recorded as absorbance in the presence of energies from 4000 to 10 000 cm^−1^ or in wavelength range from 1000 to 2500 nm for a total of 40 bulk soil samples. Each reading spectra data contained 1557 wavelength variables and as an average of 64 successive data acquisition. This spectra dataset were used for further analysis in prediction model development.
Data format	Raw Analysed Enhanced Presented as *.xls* and *.unsb* file formats
Parameters for data collection	Spectra datasets of soil samples were used to predict fertility parameters in form of soil nitrogen content (N), phosphorus (P), potassium (K), soil pH (pH), magnesium (Mg) and calcium (Ca). Soil samples were collected per 5 cm at top soil from 0 to 20 cm depth.
Description of data collection	Calibration models database were established and obtained by regressing near infrared spectroscopic data as independent variables (X) and actual soil fertility properties: N, P, K, pH, Mg and Ca as dependent variables (Y). calibration models were carried out by means of two different regression approaches namely principal component regression (PCR) and partial least square regression (PLSR). During calibration modelling, n-fold cross validation was applied to generate more robust and accurate prediction results. Cross validation can also be performed using leverage validation or test matrix methods. Moreover, generated NIR model database can be evaluated and tested using unknown independent samples datasets.
Data source location	Near infrared spectra dataset of soil samples and their fertility properties were collected at the Department of Agricultural Engineering and Department of Soil Science, *Syiah Kuala* University, Banda Aceh, Indonesia.
Data accessibility	Dataset are available on this article and can be found in Mendeley repository data: https://data.mendeley.com/datasets/h8mht3jsbz/1 or http://dx.doi.org/10.17632/h8mht3jsbz.1

## Value of the data

• Near infrared spectra datasets can be benefited for farmer communities in precision agriculture practices, especially for soil fertility monitoring.
• Calibration models database can be imported and transferred onto NIRS instrument for real time and simultaneous prediction of soil fertility properties.
• NIR spectra data and models database can be re-modelled by means of different spectra enhancement and regression algorithms to improve prediction performances.
• Spectra datasets can also be benefited in detecting hazardous contaminations on agricultural soil by re-modelling and calibrating these data with standard laboratory analysis respectively.

## 1 Data

In precision farming and agriculture practices, soil fertility is an important factor affected plant growth. Soil fertility should be monitored constantly in real time situation by determining its properties such as micro and macro nutrients, also soil mineral contents. Indeed, the development of environmental monitoring programs is very important in maintaining soil health and condition. Thus, to determine soil quality and fertility in a short time and without complicated sample preparations, near infrared reflectance spectroscopy (NIRS) is employed. Infrared spectroscopy has been widely proposed and employed as an alternative method for determining soil quality properties [1]. this technique, particularly in the form of absorbance and reflectance spectra data in the NIR region, has rapidly developed into a fast, effective and robust analytical method for many fields including in agriculture [2]. The object is irradiated with near infrared light radiation and the reaction, either in form of reflection, absorption or transmission is acquired. While the radiation penetrates into the object, its spectral characteristics changes through wavelength dependent scattering and absorption process. The contribution of each reaction depends on the chemical attributes, cell structure and physical properties of the object.

**Fig. 1:**
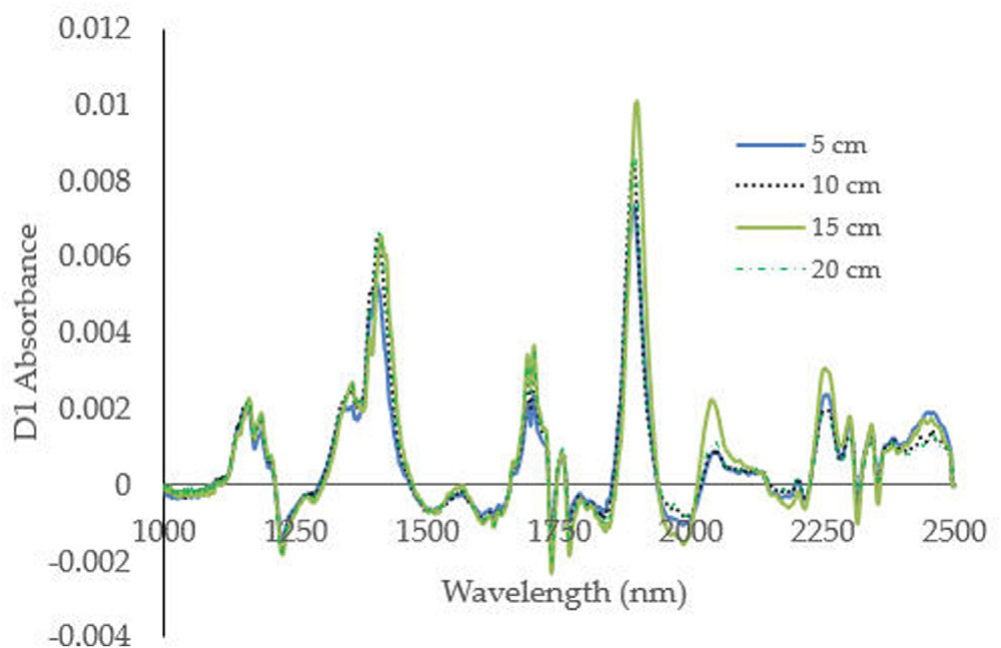
near infrared absorbance spectra after first derivative (D1) at different soil depth.

Before NIRS can be used to predict and determine soil fertility properties, spectra datasets must be acquired and calibration models database must be developed. In this datasets, soil samples were taken on top soil with from upper soil surface to maximum depth of 20 cm [3]. Spectral datasets of soil samples were measured and collected as absorbance spectrum in wavelength range from 1000 to 2500 nm as shown in Fig. 1. Absorbance spectra data for soil sample reflected the amount of light and energies absorbed by soil sample which corresponds to chemical properties such as soil nutrients and minerals content.

Soil fertility properties and other quality attributes information were buried in spectra data and can be revealed through calibration modelling by means of regression approaches. There several techniques available and commonly use in NIRS practice from which most of them are based on linear and non-linear algorithms. The two most common calibration methods are principal component regression (PCR) and partial least square regression (PLSR) [4,5]. PCR is a two-step procedure started with decomposing spectra data as X-variables by means of a principal component analysis (PCA), and then continued with fitting a multiple linear regression model, using a small number of principal components or latent variables instead of the original variables as predictors. The advantage PCR model compared to common multiple linear regression is that the X-variables (principal components) are uncorrelated, and that the noise caused during spectra data acquisition is filtered. Further, usually a small number of principal components are preferable for the effectiveness of the calibration models [6,7]. The main weakness of PCR is that the principal components are ordered according to decreasing explained variance of the spectra data matrix, from which the first principal component is used for the regression model, is not necessarily the most informative with respect to the related soil fertility attributes or other response variable.

On the other hand, partial least squares regression is an approach with close likely to PCR. The main difference of the PLSR method is that both spectra data of soil samples (X-variables) and soil fertility properties (Y-variables) are projected onto new spaces. In PLSR approach, an orthogonal basis of latent variables or factors is constructed one by one in such a way that they are oriented along the directions of maximal covariance between the spectral matrix and the response vector. Thus, PLSR algorithm ensured that latent variables are ordered according to their relevance for predicting the soil quality properties like N, P, K, pH, Mg and Ca contents. Interpretation calibration models between spectra data and soil fertility attributes is then simplified as this model is concentrated on the least possible number of latent variables.

**Fig. 2:**
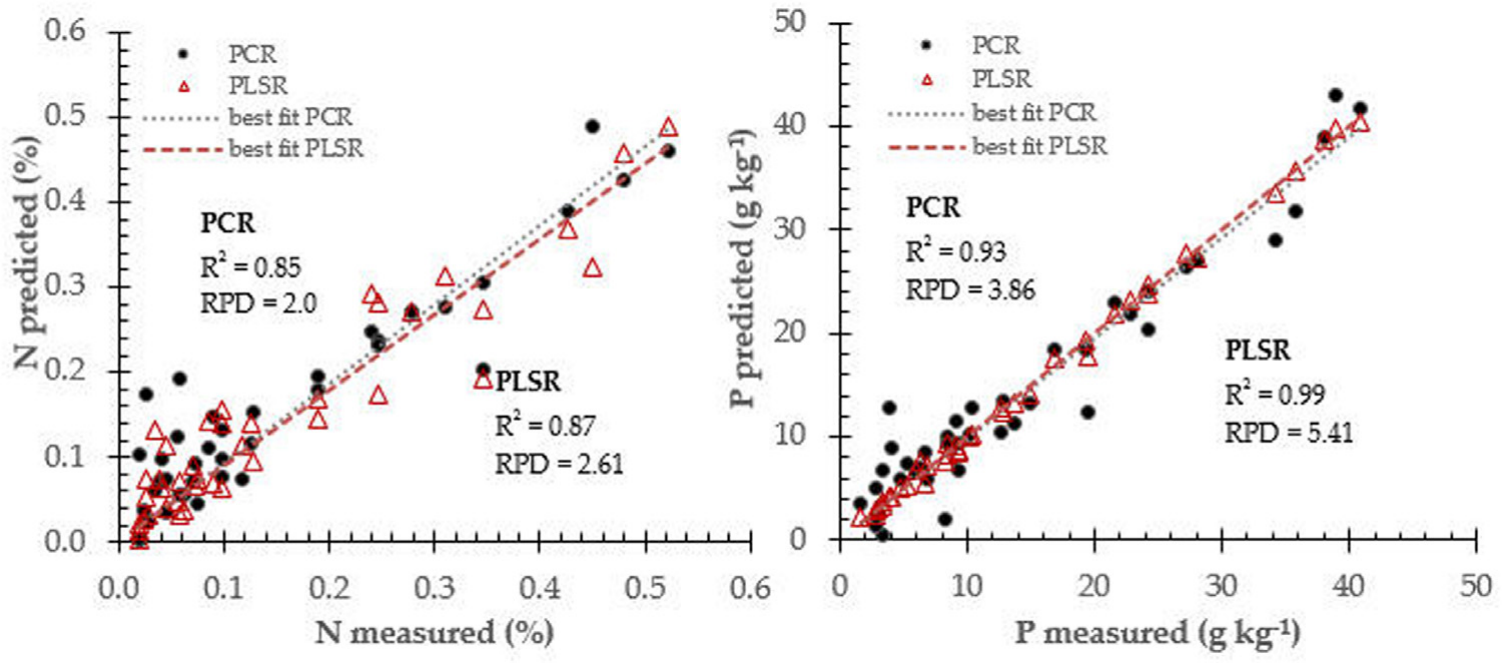
Prediction performance for soil N and P prediction using two different calibration approach: PCR and PLSR.

**Fig. 3:**
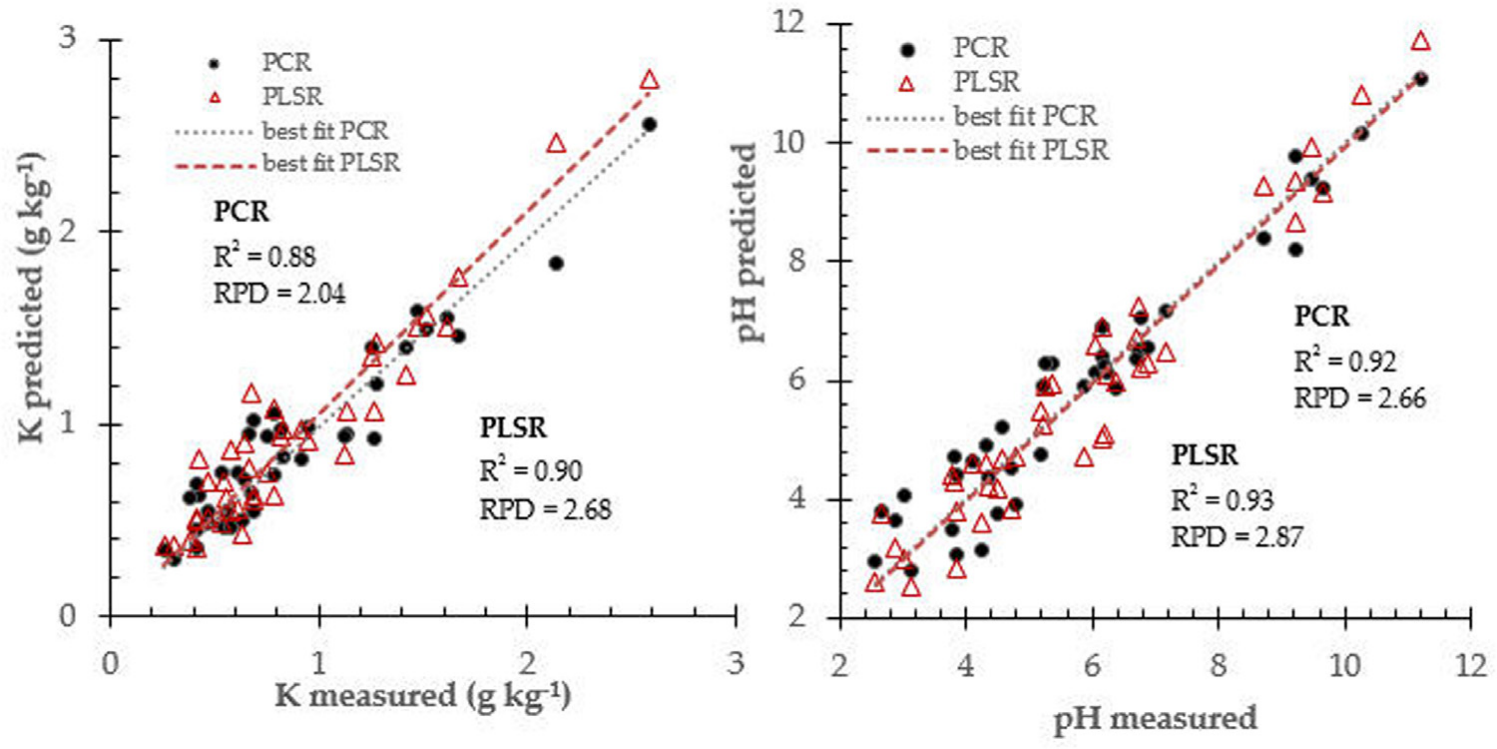
Prediction performance for soil K and pH prediction using two different calibration approach: PCR and PLSR.

**Fig. 4:**
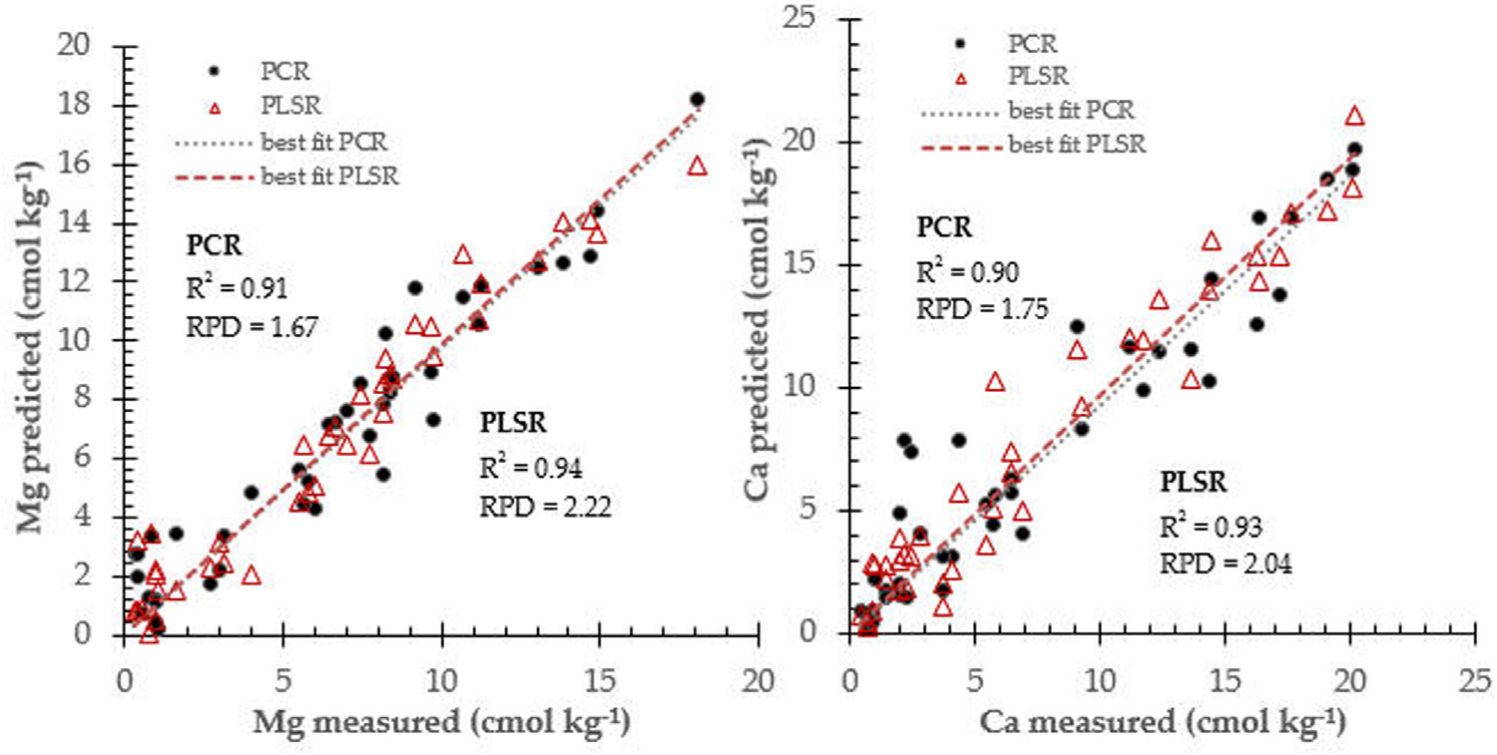
Prediction performance for soil Mg and Ca prediction using two different calibration approach: PCR and PLSR.

Calibration models database can be performed particularly well when the various X-variables express common information, for example, when there is a large data amount of correlation, or even co-linearity, like spectra data of soil samples. The required number of latent variables in PLSR algorithm is typically fewer than that in a PCR calibration model for a similar model performance. The number of latent variables required to construct calibration models is also taken into account for the model database effectiveness and to avoid model overfitting. Prediction performance using PCR and PLSR models for soil N and P prediction (see Fig. 2), for K and pH (see Fig. 3), and for Mg and Ca (see Fig. 4) respectively.

The PCR and PLSR attempted to find best correlation between near infrared spectra data and respective soil fertility properties (N, P, K, pH, Mg and Ca). they were widely employed in the near infrared spectroscopy practice and fitted in many applications [8]. Table 1 summarized prediction performance for soil fertility properties using calibration models database derived from principal component regression and partial least square algorithms.

**Table 1:** Prediction performance for all calibration models database to determine soil fertility properties.

Soil properties	Calibration models	Statistical Indicators			
		R^2^	r	RMSEC	RPD
N	PCR	0.85	0.92	0.07	2.00
	PLSR	0.87	0.93	0.04	3.50
P	PCR	0.93	0.96	2.97	3.86
	PLSR	0.99	0.99	2.12	5.41
K	PCR	0.88	0.94	0.25	2.04
	PLSR	0.90	0.95	0.19	2.68
pH	PCR	0.92	0.96	0.83	2.66
	PLSR	0.93	0.96	0.77	2.87
Mg	PCR	0.91	0.95	2.84	1.67
	PLSR	0.94	0.97	2.14	2.22
Ca	PCR	0.90	0.95	3.66	1.75
	PLSR	0.93	0.96	3.14	2.04

Ca: soil calcium, K: potassium, Mg: magnesium, N: nitrogen, P: phosphorus, pH: soil pH, PCR: principal component regression, PLSR: partial least square regression, r: correlation coefficient, R^2^: coefficient of determination, RMSEC: root mean square error in calibration, RPD: residual predictive deviation index.

Near infrared spectra data acquired from the instrument may contain spectra background information and noises which can be interfered soil quality attributes. Interfering noises, such as light scattering, path length variations and random noise due to physical sample properties or instrumental effects should be eliminated or reduced in order to obtain more reliable, robust, accurate and stable calibration models [9]. Spectra data can be enhanced in order to improve prediction accuracy and robustness of those calibration models. The most common used spectra correction methods are including: smoothing, normalization, transformation and spectra derivation.

## 2 Experimental design, materials, and methods

### 2.1 Instrument setup

Near infrared spectra data of soil samples were acquired and measured using a benchtop NIR instrument (Thermo Nicolet Antaris II) with an integrating sphere accessory. The instrument was controlled and configured under integrated software namely Thermo Integration® and Thermo Operation®. Specified tasks for spectra data acquisition were performed by establishing workflow using Thermo Integration software [10]. High resolution measurement with integrating sphere was chosen as a method for spectra acquisition. For each spectra acquisition, sample labelling was required automatically prior to spectra data collection to differ soil samples respectively.

### 2.2 Soil samples

Samples were collected as top soil samples (0-20 cm depth) from 10 different rice-paddy field and cropland sites in *Aceh Besar* district, Aceh Province. In each site, 2 soil samples were taken from 2 rice-paddy field and 2 samples from nearby cropland. Thus, a total of 40 top soil samples were collected with each sample reduced to 150 g by quartering. All soil samples were stored for a day to equilibrate, then air-dried for one week and sieved through 2 mm nylon sieve in order to remove stones, insects, large debris, pebbles and other unwanted materials [11,12]. Soil samples were then grounded in a mechanical agate grinder and passed through a 100 mesh sieve (0.150 mm diameter) [13,14]. Each bulk soil sample was equably mixed and then grouped into four sub-samples for spectra data acquisition and soil fertility properties measurements.

### 2.2 Spectra data acquisition

Near infrared (NIR) spectra data were acquired and recorded as absorbance spectra data in the presence of energies in wavenumbers 4000-10 000 cm^−1^ or in wavelength range from 1000 to 2500 nm [15,16]. Soil samples were transferred into a cylindrical quartz cup sample holder with 10 mm in depth to ensure full light penetration. The sample cylindrical cup was filled with 20 g of soil samples and levelled using a smooth edge. It was set to rotate slowly during spectra acquisition with co-added of 64 scans [17]. Soil samples were loaded into two replicate cups, each cup scanned 64 times and the two spectra averaged to account for within-sample variability and differences in packing density and particle size.

### 2.3 Soil properties data measurement

Once after spectra data acquisitions were completed, Soil quality properties (N, P, K, pH, Ca and Mg) of the soil samples were measured and determined using standard chemical laboratory methods. Soil nitrogen (N) content was determined using the *Kjeldahl* method using digestion by H_2_SO_4_ and expressed in percentage of their weight to the total weight of dry soil sample. Moreover, the soil phosphorus (P) content was determined by means of HClO_4_-H_2_SO_4_ heating extraction and a combination of molybdenum-blue colorimetric method [18,19].

The soil potassium (K) content was determined by calcining and extracting with NaOH. The K content was then measured using an atomic absorption flame photometer. On the other hand, soil pH was measured in a slurry of soil and water at ratio of 1: 2.5 respectively, by a glass electrode using an electronic pH meter. Buffer solutions with pH = 6.86 and 4.01 were used for calibrating the pH meter. Furthermore, magnesium (Mg) and calcium (Ca) content of soil samples were determined using an acid digestion method with inductively coupled plasma absorption flame spectrophotometer [17]. All chemical analyses for soil properties were carried out in duplicate and averaged. Descriptive statistics of actual measured soil fertility properties data in form of N, P, K, pH, Mg and Ca are shown in Table 2.

**Table 2:** Descriptive statistics data of actual measured soil fertility properties.

Descriptive statistics	N	P	K	pH	Mg	Ca
Mean	0.15	14.49	0.88	5.78	6.43	7.45
Max	0.52	40.92	2.58	11.21	18.07	20.18
Min	0.02	1.68	0.26	2.57	0.31	0.39
Range	0.50	39.24	2.32	8.64	17.76	19.79
Std. Deviation	0.14	11.46	0.51	2.21	4.75	6.41
Variance	0.02	131.26	0.26	4.89	22.60	41.08
RMS	0.21	18.38	1.02	6.18	7.96	9.78
Skewness	1.25	0.97	1.44	0.73	0.43	0.68
Kurtosis	0.47	-0.16	2.14	-0.12	-0.52	-0.95
Median	0.09	9.77	0.69	5.31	6.54	5.55
Q1	0.04	5.86	0.55	4.23	1.48	2.00
Q3	0.24	21.92	1.16	6.75	9.28	12.68

Ca: calcium, K: potassium, Mg: magnesium, N: nitrogen, P: phosphorus, Q1: first quartile, Q3: third quartile.

### 2.4 Sample outlier detection

**Fig. 5:**
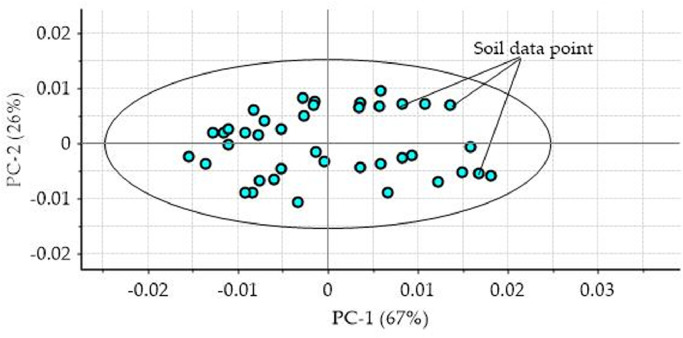
Spectra data for soil samples projected onto PCA and *Hotelling* T^2^ ellipse for outlier detection.

In order to achieve accurate and robust prediction performance, spectra data firstly can be observed and analyse for outlier detection. The most common method used to detect outliers is by projecting spectra data of soil samples onto principal component analysis (PCA) and applying *Hotelling* T^2^ ellipse respectively (see Fig. 5) [20,21]. Outliers are samples that considerably different from the other majority of remaining samples. They obviously can affect prediction performance of NIR models and should be detected prior to calibration models development. Outliers normally can be found in spectra datasets used for model calibration and validation, or arise among new samples datasets during independent prediction.

The Hotelling T^2^ ellipse is used to define statistical boundaries around the principal component analysis score plot and assuming all spectra data are distributed normally. Samples lies outside or beyond the T^2^ ellipse were to be suspected as outliers. The T^2^ ellipse was established at 95% of confidence level and it can be modified accordingly. Moreover, outliers can also be detected by means of the *Mahalanobis* distance applied with respected to raw spectra datasets. Once outliers have been identified and detected, they can be easily excluded for further analysis, but it would be better to recognize the reasons why these such samples were present and must be verified. It can help to increase the knowledge about the presented dataset and provide information whether they really acted as outliers or even leverage. Thus, improved quality of the dataset to achieve better and robust calibration model performance. Nevertheless, outliers may either present as different spectra data, or may have mistaken quality attributes measured by the reference analytical laboratory. Furthermore, outliers may remain occult until the calibration models which represented the quantitative relationship between spectra data and soil fertility properties and chemical concentration are revealed.

### 2.5 infrared spectra data corrections

Acquired spectra data may contain irrelevant background information and noises which can affect and interfere soil fertility properties and other quality attributes information. Interfering spectra data such as light scattering and other random noises due to overheated sensors, instrumental parts and changes on physical sample properties need to be reduced or even eliminated in order to obtain more accurate, robust and stable calibration models database. Therefore, it is strongly recommended to correct and enhance spectra data before establishing and constructing calibration models.

The first stage in spectra correction can be made by mean-centred (MC). This method is simply preferable due its effectively and focuses on differences between observations spectra data. Another commonly used spectra correction method is smoothing which can improve the visual aspect of the NIR spectra data. Further, normalization is also commonly used as spectra enhancement method. It can normalize data based on the mean and peak spectrum. Multiplicative scatter correction (MSC) and standard normal variate (SNV) are the two most popular spectra correction and enhancement techniques. MSC is used to compensate for additive and multiplicative effects in the spectral data caused by physical effects. This attempted to remove the effects of scattering by linearizing each spectrum to an *ideal* spectrum of the spectra data which is corresponds to the average spectrum [22,23]. On the other hand, the SNV seek to normalize each individual spectrum of the spectra data to zero mean and unit variance. Apart from the different algorithm, obtained calibration models resulted from MSC and SNC spectra data are more-less similar. Spectra corrections can also be coupled among those methods to generate better calibration and prediction performances.

### 2.6 Prediction models

Soil fertility properties and other quality attributes information are buried in the spectra data. In order to be able to reveal those information, prediction models must be established through a process called calibration. The methods adopted regression either as linear or non-linear regression approach from which spectra data (X-data) and actual measured quality parameters (Y-data) were regressed. Sample dataset used in calibration phase must be representative of the present and of future prediction samples. It means that all expected sources of variability must be considered in both calibration and validation sample datasets.

The two most common regression approaches used in calibration models development are principal component regression (PCR) and partial least square regression (PLSR). Both of them continue to be the workhorses for regression in near infrared spectroscopy applications. The initial and essences of PCR and PLSR are linear methods which assuming linear relationship of the modelled soil quality parameters and fertility properties as a function of infrared spectra data variations. PCR and PLSR requires latent variables (LVs) in constructing calibration models which known as principal component (PC) in PCR, and factors in PLSR respectively.

**Fig. 6:**
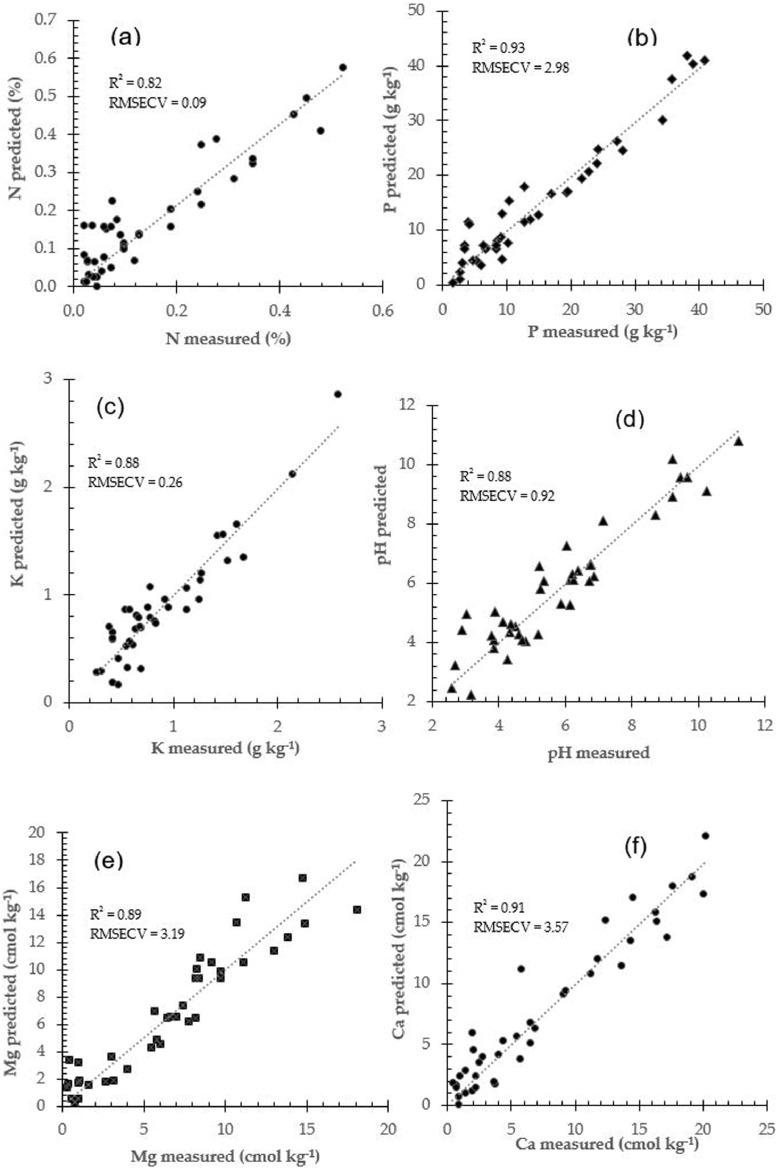
Prediction performance of calibration models to predict soil fertility properties.

Established calibration models database need to be evaluated and quantified by means of validation, either as cross validation or independent validation. Prediction performances can be evaluated using these following statistical parameters: the coefficient of determination (R^2^), coefficient of correlation (r) between predicted and measured soil fertility properties, prediction error which is defined as the root mean square error in calibration (RMSEC), root mean square error in cross validation (RMSECV), and the residual predictive deviation (RPD), defined as the ratio between standard deviation (SD) of the actual measured fertility parameters (N, P, K, pH, Mg and Ca), and the RMSE of respective predicted soil properties. The higher value of RPD, the greater probability of models to predict quality parameters or chemical concentrations of soil samples dataset accurately (see Fig. 6).

Beside using linear regression approaches, calibration models database can also be constructed using non-linear regression methods such support vector machine regression (SVMR) or artificial neural networks (ANN) regression based. Both SVMR and ANN are more flexible calibration methods since they can handle linear and nonlinear relationship between the near infrared spectra data and corresponding soil fertility parameters or other chemical constituents of biological materials.

Support vector machine (SVM) is a particular class of algorithm, characterized by the use of kernels in its regression and classification approaches. At the beginning of initial development, the SVM method was normally employed for classification problems, but nowadays it also has been applied for regression purposes. In kernel-based methods like SVM, calibration model is carried out in a space of non-linearly transformed input data and it defined by the kernel function. On the other hand, artificial neural network (ANN) is an algorithm which is inspired to mimic human brain that is characterized by its analogy with our biological neuron. The ANN typically consists of three layers called as input layer, hidden layer and output layer. Like our brains, each input is connected with cells called neurons. Every neuron of the input layer is connected to every neuron of the hidden layer, and every neuron of the hidden layer is connected to the output layer.

Calibration models based on ANN simulates the biological neuron by multiplication of the input signal or spectra data (X-data) with the synaptic weight (W) to derive the output signal or soil fertility properties (Y-data). A neuron is acted as a computational device that calculates the weighted sum of its input and calculates the output signal from this using a non-linear function. For the efficiency and simplification purposes, spectra data of near infrared spectroscopy can be subjected firstly onto the principal component analysis, then, score value of first seven latent variables were used as input data instead of all infrared spectra values. Generally, calibration models database generated from the non-linear regression approaches achieved better prediction performances than linear regression one.

At this point, the near infrared spectroscopy is obviously can be employed to determine soil fertility parameters and certainly can be used in many field of multidisciplinary area of research and applications. The five pillars of NIRS comprise the skills of spectroscopy, calibration modelling or called as *chemometrics*, NIR instruments design and construction, standard chemical analysis and data transformation. All of them simultaneously necessary to make proper use of infrared technology in agricultural applications.

## Declaration of Competing Interest

The authors declare that they have no known competing financial interests or personal relationships which have, or could be perceived to have, influenced the work reported in this article.

## Appendix Supplementary materials

Image, application 1
